# Diagnostic performance of the IMMY cryptococcal antigen lateral flow assay on serum and cerebrospinal fluid for diagnosis of cryptococcosis in HIV-negative patients: a systematic review

**DOI:** 10.1186/s12879-023-08135-w

**Published:** 2023-04-06

**Authors:** Catriona Macrae, Jayne Ellis, Suzanne H. Keddie, Jane Falconer, John Bradley, Ruth Keogh, Oliver Baerenbold, Heidi Hopkins, Joseph N. Jarvis

**Affiliations:** 1 Infectious Diseases Unit, NHS Lanarkshire, University Hospital Monklands, Monkscourt Avenue, Airdrie, ML6 0JS UK; 2 London School of Hygiene and Tropical Medicine, Keppel Street, London, WC1E 7HT UK; 3 Infectious Diseases Institute, Makerere University, PO Box 22418, Kampala, Uganda; 4 Botswana Harvard AIDS Institute Partnership, Gaborone, Botswana

**Keywords:** Cryptococcosis, HIV-negative, CrAg, Cryptococcal antigen, Lateral flow assay, Diagnosis, Diagnostic, Diagnostic performance, Serum, CSF

## Abstract

**Background:**

The incidence of cryptococcosis amongst HIV-negative persons is increasing. Whilst the excellent performance of the CrAg testing in people living with HIV is well described, the diagnostic performance of the CrAg LFA has not been systematically evaluated in HIV-negative cohorts on serum or cerebrospinal fluid.

**Methods:**

We performed a systematic review to characterise the diagnostic performance of IMMY CrAg® LFA in HIV-negative populations on serum and cerebrospinal fluid. A systematic electronic search was performed using Medline, Embase, Global Health, CENTRAL, WoS Science Citation Index, SCOPUS, Africa-Wide Information, LILACS and WHO Global Health Library. Studies were screened and data extracted from eligible studies by two independent reviewers. A fixed effect meta-analysis was used to estimate the diagnostic sensitivity and specificity.

**Results:**

Of 447 records assessed for eligibility, nine studies met our inclusion criteria, including 528 participants overall. Amongst eight studies that evaluated the diagnostic performance of the IMMY CrAg^®^ LFA on serum, the pooled median sensitivity was 96% (95% Credible Interval (CrI) 68–100%) with a pooled specificity estimate of 96% (95%CrI 84–100%). Amongst six studies which evaluated the diagnostic performance of IMMY CrAg^®^ LFA on CSF, the pooled median sensitivity was 99% (95%CrI 95–100%) with a pooled specificity median of 99% (95%CrI 95–100%).

**Conclusions:**

This review demonstrates a high pooled sensitivity and specificity for the IMMY CrAg^®^ LFA in HIV-negative populations, in keeping with findings in HIV-positive individuals. The review was limited by the small number of studies. Further studies using IMMY CrAg^®^ LFA in HIV-negative populations would help to better determine the diagnostic value of this test.

**Supplementary Information:**

The online version contains supplementary material available at 10.1186/s12879-023-08135-w.

## Background

Cryptococcosis is a fungal infection caused by the pathogenic *Cryptococcus* species, of which there are seven recognised species: *C. neoformans* variety *grubii*, *C. neoformans* variety *neoformans* and five species within *C. gatti* [1]. Infection occurs following inhalation of fungal cells which may lead to either asymptomatic colonisation or pulmonary cryptococcal disease [2] presenting with cough, fever, shortness of breath and/or pulmonary nodules on chest radiographs [3]. *Cryptococcus spp.* may disseminate to cause cryptococcal antigenaemia, with or without progression to multi-organ disease. Dissemination to the central nervous system causes cryptococcal meningitis, which typically presents with fever, headache, neck stiffness, altered mental status and visual disturbance [2]. Other body sites such as liver, spleen skin and bone are less commonly affected [3].

Cryptococcal infection most often occurs in people living with HIV (PLWH); however, the proportion of cases in HIV-negative patients is increasing in high income countries [4, 5], in part due to increasing use of immunosuppressive therapies for cancer chemotherapy and organ transplantation [4, 6].

In addition to immunosuppressive therapy or solid organ transplantation, hematopoietic and other malignancies, innate immune defects, advanced renal or liver disease, diabetes mellitus, rheumatologic diseases and sarcoidosis increase the risk of cryptococcal infection [3, 7, 8]. Clinical cases of cryptococcal disease have also been reported in apparently immunocompetent individuals [2, 3, 9].

Cryptococcal antigen (CrAg) is a biomarker of cryptococcosis, and detection of CrAg in cerebrospinal fluid (CSF), serum, plasma or whole blood either by lateral flow assay (LFA), latex agglutination (LA) or enzyme-linked immunosorbent assays (ELISA) is the cornerstone in diagnosing cryptococcosis. Other diagnostic modalities include basic CSF analysis (white cell count, protein, glucose), India ink staining, cryptococcal culture on Sabouraud’s dextrose agar, and histology. Multiplex polymerase chain (PCR) platforms including *Cryptococcus spp*. as a target pathogen have also been evaluated as a diagnostic tool for cryptococcosis; and matrix-assisted laser desorption ionization–time-of-flight mass spectrometry (MALDI-TOF) has also been reported to detect *Cryptococcus spp.* in clinical specimens [10]. The World Health Organization (WHO) recommends rapid Ag-detection assays for diagnosis of cryptococcal disease in PLWH [11].

The IMMY CrAg^®^ LFA (Norman, Oklahoma, USA), approved by the U.S. Food and Drug Administration (FDA) in 2011, is an immunochromatographic dipstick assay that detects antigen with qualitative or semiquantitative results. The IMMY CrAg^®^ LFA is currently the most sensitive commercially available cryptococcal diagnostic test, with superior sensitivity to India ink microscopy on CSF, CSF cryptococcal culture, Meridian Cryptococcal Antigen Latex Agglutination System (CALAS^®^), the Meridian EIA assay, and the BioFire^®^ FilmArray^®^ Meningitis/Encephalitis (ME) panel [12–16]. The IMMY CrAg^®^ LFA was therefore employed as part of The Febrile Illness Evaluation in a Broad Range of Endemicities (FIEBRE) study; a prospective observational study to investigate the infectious causes of fever at four sites in Africa and Asia, collecting data and samples from PLWH and HIV-negative inpatients and outpatients [17]. FIEBRE focused on illnesses deemed preventable or treatable, of which cryptococcosis is an important example. Lumbar punctures were not routinely conducted as part of the FIEBRE diagnostic package, so the IMMY CrAg^®^ LFA performed on serum samples was chosen as the diagnostic strategy for all FIEBRE participants.

The performance of CrAg testing for the diagnosis of cryptococcosis in HIV-negative populations has not previously been systematically reviewed. This review aims to assess diagnostic performance of the IMMY CrAg^®^ LFA compared to other cryptococcal diagnostic tests for the diagnosis of cryptococcosis in HIV-negative persons.

## Methods

This systematic review was registered at PROSPERO (www.crd.york.ac.uk/PROSPERO) as CRD42022314040 on 02/03/2022 and is reported following the Preferred Reporting Items for Systematic Reviews and Meta-Analyses (PRISMA) statement for the reporting of systematic reviews and meta-analyses [18].

### Literature search strategy

The following searches were conducted with an aim of identifying all studies reporting on the diagnostic performance of the IMMY CrAg^®^ LFA for the diagnosis of cryptococcosis in HIV-negative populations. The study population was HIV-negative adults and children. The index test was the IMMY CrAg^®^ LFA and comparator tests were any alternative cryptococcal diagnostic test/s, including clinical composite end-points.

A systematic electronic search was conducted using Medline, Embase, Global Health, CENTRAL, WoS Science Citation Index, SCOPUS, Africa-Wide Information, LILACS and WHO Global Health Library. A draft search strategy was compiled in the OvidSP Medline database by an experienced information specialist (JF). The search strategy included strings of terms, synonyms and controlled vocabulary terms (where available) to reflect two concepts: *Cryptococcus spp.* and IMMY lateral flow assay. Further information on the search methodology is available in Additional file 1.

### Information management

All citations identified were imported into EndNote™ X9 software (Pennsylvania, PA, USA). Duplicates were identified and removed using the method described on the London School of Hygiene & Tropical Medicine Library & Archives Service blog [19].

The OvidSP MEDLINE search was adapted for each of the bibliographic databases. The search period was 2009–July 2021, as the IMMY CrAg^®^ LFA was introduced in 2009.

### Study selection

A two-stage screening process was employed: (1) at title and abstract and (2) at full-text level according to eligibility criteria as detailed below. Screening was performed in duplicate independently by two reviewers (CM, JE), and any disagreements were resolved by discussion. Reports not meeting the eligibility criteria were excluded. Reference and citation checking were conducted for included articles.

Studies were eligible for inclusion if they reported on the use of the IMMY CrAg^®^ LFA tested on serum and/or CSF, in HIV-negative persons, compared to any other test/s or composite used to diagnose cryptococcal disease. Studies including asymptomatic and/or symptomatic persons were included. We included all study types, irrespective of country, region, continent, or level of care (primary, secondary, or tertiary). Studies that did not have disaggregate data for HIV-negative participants were excluded.

Study selection criteria

  

1. Paper written in English,
2. Studies from 2009 onwards,
3. Study reports use of IMMY CrAg® LFA on serum or CSF,
4. Samples tested are from HIV-negative persons (adults and children) only, or if HIV-positive persons included disaggregated data is presented,
5. Paired data: The same samples tested with IMMY CrAg® LFA compared to any reference standard,
6. Not case study or case report (and participants tested n>5),
7. Full peer-reviewed published text available.

### Data extraction and synthesis

For all eligible studies two reviewers (CM, JE) independently extracted data using an Excel spreadsheet (Microsoft Corp., Redmond, WA, USA) including sample size, study design, participant characteristics, sample characteristics, flow and timing of sample analysis and comparator test characteristics. For each study the performance results for the CrAg LFA test (Index, “I”) and the comparator test (“C”) were extracted into 2 × 2 tables. In studies using multiple comparator tests a 2 × 2 table was generated for each comparator.

### Quality assessment

Two reviewers (CM, JE) used the QUADAS-2 (quality assessment of diagnostic-accuracy studies-2) tool for quality assessment to evaluate the risk of bias and applicability of all included studies [20]. Disagreements were resolved by discussion.

### Statistical analysis and data synthesis

The original analysis plan included random effect meta-analysis. A random-effect meta-analysis accounting for between-study heterogeneity would usually be the model of choice in this scenario because we would not expect the sensitivity and specificity of the diagnostic test to be the same in each study. In this systematic review however, there were a limited number of studies and only one study that actively sought to investigate the specificity [21] of the diagnostic test of interest, making a random-effects model inappropriate [22]. A fixed-effect model was therefore used in this instance.

Modelled estimates for the sensitivity and specificity of the IMMY CrAg^®^ LFA were calculated for each study, as well as a single pooled estimate. The studies were subdivided by sample type; the estimates were calculated for use of the CrAg LFA on both serum and CSF. For the analysis similar comparator tests, for example, different latex agglutination tests, were grouped to represent a single comparator test.

We chose to fit a Hierarchical Summary Receiver Operating Characteristic (HSROC) model [23] with fixed accuracy and threshold parameters. This model still ensures that sensitivity and specificity are jointly estimated as well as accounting for imperfect reference tests [24] while also allowing for asymmetry in the SROC curve. This model can be seen as a simplification of the random effects model fit in Jullien et al. [25] where the variances of the random effects are zero $(i.e. {\sigma }_{\theta }=0, \mathrm{and}\, {\sigma }_{\alpha }=0, \,\mathrm{such\,that} {\uptheta }_{j}\, \mathrm{and}\, {\alpha }_{j}\, \mathrm{are\,equal\,to}\,\Theta\, \mathrm{and}\,\Lambda\,, {\mathrm{respectively}})$.

All analyses were conducted in R with stan [26]. For model code see: https://github.com/shk313/diagnostic-test-metaanalysis/tree/main/CrAg.

## Results

**Fig. 1:**
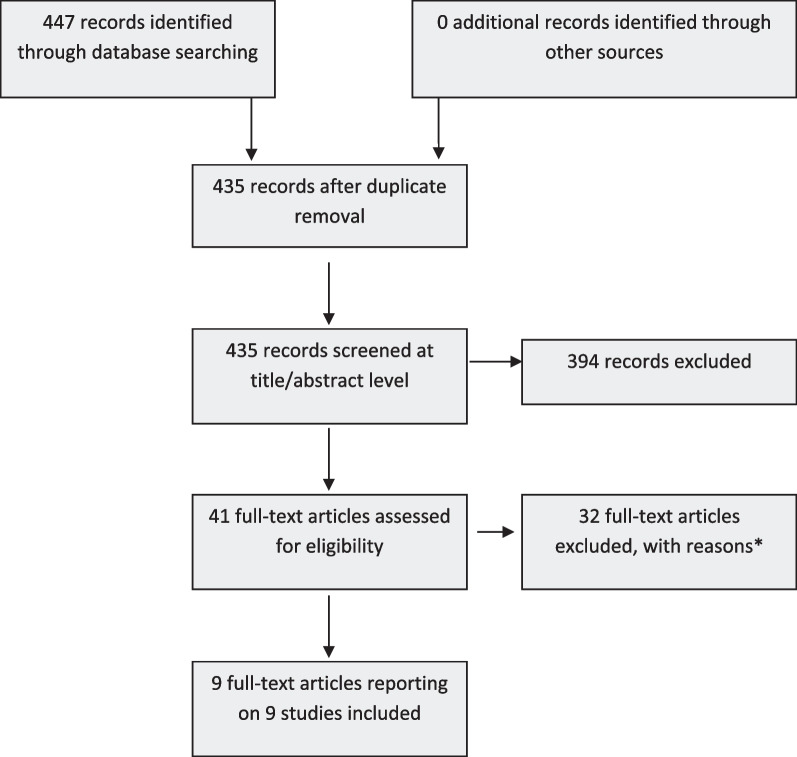
PRISMA diagram showing selection of studies for a systematic review of the diagnostic performance of the IMMY cryptococcal antigen lateral flow assay on serum and cerebrospinal fluid in HIV-negative patients. *No full text available (6), paper not written in English (2), samples tested not from HIV-negative persons (adults or children) – or no disaggregate data for HIV-negative persons (13), study reports on < 4 cases (8), does not report use of IMMY CrAg^®^ LFA on serum or CSF (3)

Our searches yielded 447 potentially eligible articles. After removal of duplicates (n = 12), screening of titles and abstracts (n = 435) and review of the full texts (n = 41), nine articles met our eligibility criteria for inclusion (Fig. 1 PRISMA diagram).

### Study description

The nine articles included and their key characteristics are summarised in Tables 1 and 2. In total, the included studies evaluated the diagnostic performance of the IMMY CrAg^®^ LFA in 528 HIV-negative persons, across three continents. The reports were published between 2015 and 2021. The mean number of participants per study was 59, with a median of 37 participants per study. The age of study participants ranged from eight [27] to 88 years. [28, 29] The majority of participants were male, with the percentage of female participants ranging from 27 to 50% [28, 30]. All studies used cross sectional study design.

**Table 1:** Summary of characteristics of studies reporting findings of IMMY CrAg^®^ lateral flow assay on serum and comparator test(s)

	Author, date of publication	Setting	n	Study population	Immunosuppressed n (%)	Cryptococcal disease phenotype	Samples tested (n)	Comparator test(s)	Reported sensitivity (95% CI)	Reported specificity
1	Dubbels 2017	USA	37	Not reported	15 (40.5%)	Cryptococcal meningitis, pulmonary cryptococcosis, other	36	Culture, histology, LA, composite^c^	Not calculated	66%
2	Harrington 2021	USA	96	Asymptomatic inpatients and outpatients	43 (45%)	No disease	35	LA	Not calculated	Not calculated
3	Hevey 2020	USA	34	Symptomatic inpatients	Not reported	Pulmonary cryptococcosis, other^b^	34	Composite clinical and laboratory end point^d^	Overall 85.3%, localised pulmonary 90.9% (58.7–99.8%), disseminated 82.6% (61.2–95.1%)	Not calculated
4	Jitmuang 2015	USA	31	Symptomatic inpatients	17 (55%)	Cryptococcal antigenemia,^a^ Cryptococcal meningitis, pulmonary cryptococcosis, other^b^	53	LA, EIA	100% (92–100%)	Not calculated
5	Min 2020	China	78	Symptomatic inpatients	17 (22%)	Pulmonary cryptococcosis	78	Lung biopsy (histopathology)	69.2% overall, immunocompetent 80.3%, immunocompromised 29.4%	Not calculated
6	Tintelnot 2015	Germany	8	Not reported^g^	0	Cryptococcal antigenemia,^a^ cryptococcal meningitis	9	LA	Not calculated	Not calculated
7	Wang 2020	China	149	Symptomatic inpatients	55 (37%)	Cryptococcal antigenemia,^a^ cryptococcal meningitis, pulmonary cryptococcosis	136	Composite clinical and laboratory end point^e^	Titre 1:10 39.6% (29.7–50.1%), Titre 1:5 72.9% (62.9–81.5%)	Titre 1:10 100%(69.2–100%), Titre 1:5 70.0% (34.8–93.3%)
8	Wu 2020	China	37	Symptomatic and asymptomatic inpatients	15 (41%)	Pulmonary cryptococcosis	25	Composite clinical and laboratory end point^f^	Not calculated	Not calculated

Cryptococcal disease phenotypes

^a^Cryptococcal antigenaemia: isolated cryptococcal antigenaemia without evidence of disease

^b^Other: any other cryptococcal disease including disseminated disease

Definition of composites used as comparators

^c^(i) a Cryptococcus species was recovered in culture from any specimen source, (ii) a Cryptococcus species was histopathologically identified in any specimen, or (iii) the patient responded to targeted antifungal therapy with concomitant decreases in serial CrAg LFA titers [28]

^d^Positive serum or CSF CrAg, isolation of Cryptococcus neoformans in culture, or identification by the International Classification of Diseases (ICD) 9th (117.5, 321.0) or 10th (B45.1-B45.9) editions [34]

^e^Cryptococcal infections defined as: either “proven”, “probable”, “possible” or “non-crypto- coccosis”, as described for other invasive fungal diseases, with some modifications in patients with low CrAg LFA titers as per De Pauw et al. [36]

^f^Proven pulmonary cryptococcosis based on the following criteria: histopathological, cytopathological or direct microscopic examination of a specimen obtained by a needle aspiration or biopsy from a normally sterile site (other than mucous membranes) showing encapsulated budding yeasts; or probable PC if each of the three elements of host factor, clinical features and mycological evidence were present [33]

^g^Testing of stored laboratory samples

**Table 2:** Summary of characteristics of studies reporting findings of IMMY CrAg^®^ lateral flow assay on cerebrospinal fluid (CSF) and comparator test(s)

	Author, date of publication	Setting	n	Study population	Immunosuppressed n (%)	Cryptococcal disease phenotype	Samples tested (n)	Comparator test(s)	Reported sensitivity (95% CI)	Reported specificity
1	Chen 2016	China	58	Symptomatic inpatients	7 (12%)	Cryptococcal meningitis	85	India ink, culture, LAMP, qPCR	97.6%, (91.8–99.7%)	Not calculated
2	Dubbels 2017	USA	37	Not reported	15 (40.5%)	Cryptococcal meningitis, pulmonary cryptococcosis, other	12	Culture, histology, LA, composite^c^	Not calculated	66%
3	Harrington 2021	USA	96	Asymptomatic inpatients and outpatients	43 (45%)	No disease	79	LA	Not calculated	Not calculated
4	Jitmuang 2015	USA	31	Symptomatic inpatients	17 (55%)	Cryptococcal antigenemia,^a^ Cryptococcal meningitis, pulmonary cryptococcosis, other^b^	11	LA, EIA	100% (66–100%)	Not calculated
5	Tintelnot 2015	Germany	8	Not reported^e^	0	Cryptococcal antigenemia,^a^ cryptococcal meningitis	2	LA	Not calculated	Not calculated
6	Wang 2020	China	149	Symptomatic inpatients	55 (37%)	Cryptococcal antigenemia,^a^ cryptococcal meningitis, pulmonary cryptococcosis	22	Composite clinical and laboratory end point^d^	CSF titre 1:10 50.0% (21.1–78.9%), CSF titre 1:5 66.7% (34.9–90.1%)	Not calculated

Cryptococcal disease phenotypes

^a^Cryptococcal antigenaemia: isolated cryptococcal antigenaemia without evidence of disease

^b^Other: any other cryptococcal disease including disseminated disease

Definition of composites used as comparators

^c^(i) a Cryptococcus species was recovered in culture from any specimen source, (ii) a Cryptococcus species was histopathologically identified in any specimen, or (iii) the patient responded to targeted antifungal therapy with concomitant decreases in serial CrAg LFA titers [28]

^d^Cryptococcal infections defined as: either “proven”, “probable”, “possible” or “non-cryptococcosis”, as described for other invasive fungal diseases, with some modifications in patients with low CrAg LFA titers as per De Pauw et al. [42]

^e^Testing of stored laboratory samples

In seven of the nine included studies, a proportion (12–55%) of the participants were reported to be immunosuppressed. Where documented, immunosuppression included long-term immunosuppressive therapy (1–20%) [21, 27, 28, 31], solid organ transplant (3–19%) [21, 27, 28, 31], malignancy (3–11%) [21, 28, 29, 31–33], innate immune defects (3–39%) [27, 32], liver disease (1–19%) [21, 27, 32], renal disease (3%) [32], diabetes (3–14%) [27–29, 32, 33] and rheumatological disease (1–14%) [21, 27, 33]. Other forms of reported immunosuppression included tuberculosis [27] and myasthenia gravis [32].

The majority of studies (six of seven) reported on the diagnostic performance of the IMMY LFA amongst symptomatic inpatients. This included patients with a range of cryptococcosis clinical phenotypes including cryptococcal antigenaemia (n = 56), cryptococcal meningitis (n = 103), pulmonary cryptococcosis (n = 233), and other cryptococcal disease including unspecified disseminated cryptococcosis (n = 39). There was significant heterogeneity between study cohorts with one study looking at cryptococcal meningitis only [27], two looking at only pulmonary cryptococcosis [31, 33], and the others including a combination of cryptococcal meningitis, pulmonary cryptococcosis and cryptococcal antigenaemia in varying proportions [28–30, 32, 34].

The diagnostic performance of the IMMY CrAg^®^ LFA was compared to a wide range of comparators. Across all studies the results of IMMY CrAg^®^ LFA testing on serum were compared with eight different cryptococcal diagnostic tests/composites: IMMY LA (n = 1), Meridian LA (n = 3), Biorad LA (n = 1) and Remel LA (n = 1) [21, 30, 32], Meridian EIA (n = 1) [32], culture of any site (n = 1) [28], histopathology (n = 2) [28, 31], and composites (n = 3) [29, 33, 34]. IMMY CrAg^®^ LFA testing on CSF was compared with 10 different cryptococcal diagnostic tests/composites: IMMY LA (n = 1), Meridian LA (n = 3), Biorad LA (n = 1) and Remel LA (n = 1) [21, 30, 32], Meridian EIA (n = 1) [32], culture (n = 2) [27, 28], microscopy performed on India ink-stained samples (n = 1) [27], composites (n = 2) [28, 29], LAMP (n = 1) [27] and qPCR (n = 1) [27]. A total of three clinical composite end-point definitions were used, as described in the footnotes of Tables 1 and 2 [28, 33, 34].

### Findings

Amongst eight studies which used the IMMY CrAg^®^ LFA on serum to detect cryptococcal disease, the pooled sensitivity estimate, as compared to all comparator tests, was calculated as 96% (95%CrI 68–100%) and the pooled specificity estimate was calculated as 96% (95%CrI 84–100%). Amongst six studies which evaluated the diagnostic performance of IMMY CrAg^®^ LFA on CSF, the pooled sensitivity was calculated as 99% (95%CrI 95–100%) and pooled specificity 99% (95%CrI 95–100%).

**Fig. 2:**
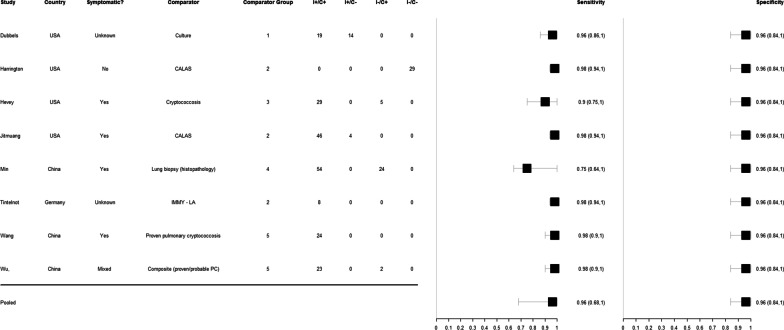
Forest plot of IMMY CrAg^®^ lateral flow assay sensitivity and specificity on serum

**Fig. 3:**
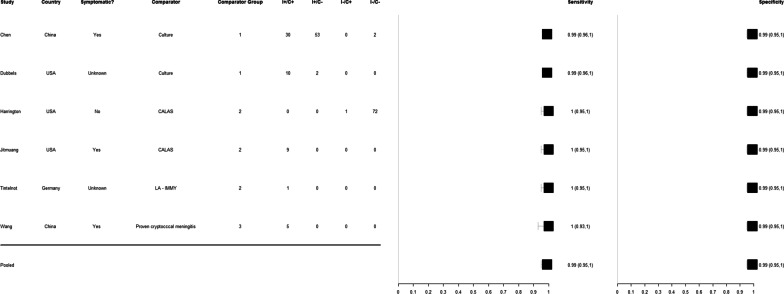
Forest plot of IMMY CrAg^®^ lateral flow assay sensitivity and specificity on cerebrospinal fluid

The estimated sensitivity and specificity of the IMMY CrAg^®^ LFA in each study as well as the pooled estimates from testing on serum and CSF are shown in Figs. 2 and 3.

### Methodological quality of included studies

Table 3 summarises the risk of bias and applicability concerns for each study. Overall there were no concerns about the applicability of the included studies. All studies were classified as having some risk of bias however, either with respect to (i) patient selection, (ii) interpretation of the index test, (iii) choice and/or interpretation of the reference standard, or (iv) sample flow and timing. In seven of nine studies, bias concerns were raised in ≥ 2 of the categories; three studies were classified as being at high risk of bias. The primary risk of bias category highlighted was in relation to interpretation of the index test, as in seven studies it was unclear whether the IMMY CrAg^®^ LFA result was interpreted in isolation, without prior knowledge of the results of the comparator test/s. Additionally, four studies were classified as being an unclear risk of bias with respect to patient selection, because it was not reported if patient sampling was random and/or whether the study avoided inappropriate exclusions.

**Table 3:**
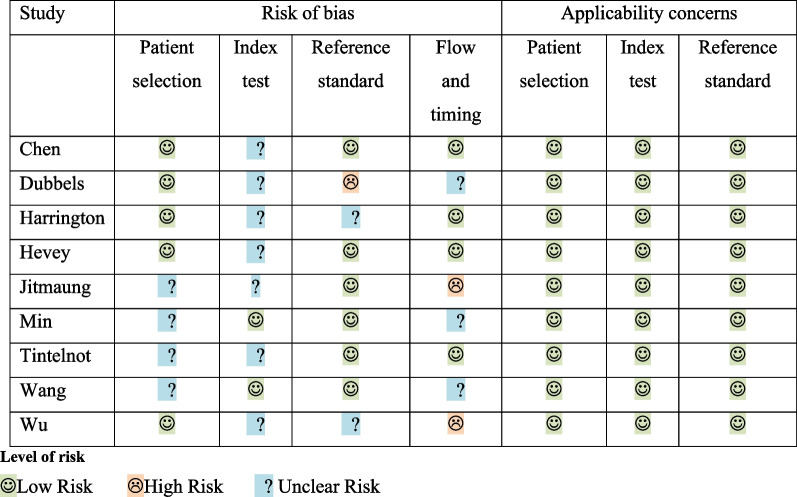
Quality assessment evaluating the risk of bias and applicability of all included studies using the QUADAS-2 (Quality Assessment of Diagnostic Accuracy Studies) tool

QUADAS-2 Scoring System

Domain 1: patient selection

Risk of bias: could the selection of patients have introduced bias? Signalling question 1: was a consecutive or random sample of patients enrolled? Signalling question 2: was a case–control design avoided? Signalling question 3: did the study avoid inappropriate exclusions?

Applicability: are there concerns that the included patients and setting do not match the review question?

Domain 2: index test risk of bias: could the conduct or interpretation of the index test have introduced bias? Signalling question 1: Were the index test results interpreted without knowledge of the results of the reference standard? Signalling question 2: If a threshold was used, was it pre-specified?

Applicability: are there concerns that the index test, its conduct, or interpretation differ from the review question?

Domain 3: reference standard risk of bias: could the reference standard, its conduct, or its interpretation have introduced bias? Signalling question 1: is the reference standard likely to correctly classify the target condition? Signalling question 2: were the reference standard results interpreted without knowledge of the results of the index test?

Applicability: are there concerns that the target condition as defined by the reference standard does not match the question?

Domain 4: flow and timing risk of bias: could the patient flow have introduced bias? Signalling question 1: was there an appropriate interval between index test and reference standard?

Signalling question 2: did all patients receive the same reference standard?

Signalling question 3: were all patients included in the analysis?

## Discussion

In this review, we evaluated the diagnostic performance of the IMMY CrAg^®^ LFA for diagnosis of cryptococcal disease amongst 528 HIV-negative persons from 9 studies. The point estimate for the sensitivity and specificity of the IMMY CrAg^®^ LFA from the pooled values were good in both serum and CSF (> 95% for both), in keeping with estimates reported in HIV positive cohorts [12, 13, 15, 16]. This is an important finding because, although the greatest burden of cryptococcal disease occurs in PLWH, this globally endemic fungal pathogen also infects HIV-negative individuals in increasing proportions.

These findings are consistent with the diagnostic accuracy literature from HIV-positive cohorts. In all published studies in PLWH, the IMMY CrAg^®^ LFA has been found to be more sensitive than all other cryptococcal diagnostic tests. In a large multi-site validation study amongst PLWH in Uganda and South Arica, the IMMY CrAg^®^ LFA performed on CSF was more sensitive than CSF culture (99.3% vs 90.0%), and more sensitive and specific than India ink microscopy on CSF (99.3% vs 86.1% and 99.1% vs 97.3% respectively) [12]. A study comparing IMMY CrAg^®^ LFA to Meridian Cryptococcal Antigen Latex Agglutination System (CALAS^®^) and Meridian enzyme immunoassay (EIA), which tested 1,000 specimens (589 serum and 411 CSF) in parallel demonstrated higher sensitivity of the IMMY CrAg^®^ LFA due to improved sensitivity for serotype C Glucuronoxylomannan (GXM) [13]. Similarly, the IMMY CrAg^®^ LFA has better diagnostic performance than current PCR-based cryptococcosis diagnostics [15]. Amongst 328 adult and 42 paediatric CSF specimens evaluated using a multiplex PCR-based commercial assay (the BioFire^®^ FilmArray^®^ Meningitis/Encephalitis (ME) panel; BioFire Diagnostics, Salt Lake City, Utah, USA), for *Cryptococcus spp*., sensitivity was 82% and specificity was 98%, using CSF CrAg testing as the reference standard [15].

A systematic review and meta-analysis of 11 studies compared CrAg testing, of serum or CSF, to CSF microscopy with India ink staining, and CSF culture for the diagnosis of cryptococcal meningitis in symptomatic PLWH [16]. In all studies fungal culture was the reference standard for confirming cryptococcal meningitis. The review calculated the sensitivity and specificity of both LA and LFA CrAg tests on serum and CSF, using pooled data from multiple studies. For LA on serum (five diagnostic cohorts, 256 participants) the pooled sensitivity estimate was 100% (99.5–100) with pooled specificity estimate 96.7% (93.8–98.9). For LFA on serum (three diagnostic cohorts, 1690 participants) the pooled sensitivity estimate was 97.9% (87.9–100) and pooled specificity estimate was 89.5% (74.3–98.5). LA showed similar sensitivity in serum as LFA (*P* = 0.08) and there was no statistically significant difference in specificity (*P* = 0.14). For LA on CSF (10 diagnostic cohorts, 1810 participants) the pooled sensitivity was 97.1% (91.9–99.0) and pooled specificity was 99.1% (93.8–99.9). For LFA on CSF (6 diagnostic cohorts, 3099 participants) the pooled sensitivity was 99.5% (97.2–99.9) and pooled specificity was 99.5% (94.2–99.9). There was some evidence that LFA may have better sensitivity in CSF (*P* = 0.07) than LA but specificities were comparable (*P* = 0.54) [16]. From our analysis the high sensitivity and specificity of IMMY CrAg^®^ LFA in serum and CSF of HIV-uninfected people is in keeping with previously reported values in studies of CrAg testing on PLWH.

There were several limitations to our review. Firstly, due to the lack of data on performance of IMMY CrAg^®^ LFA in HIV-negative people, only nine studies reporting results from a total of 528 participants were suitable for inclusion in the review. Amongst these studies, the diverse patient characteristics, range of comparator tests and cryptococcal disease phenotype made comparison difficult. The majority of studies recruited symptomatic patients or tested samples of patients known to have cryptococcal disease, with only one study screening asymptomatic patients. This limited the statistical analysis as there were very few negative IMMY CrAg^®^ LFA results in the 2 × 2 tables. For this reason, a fixed effect meta-analysis was used. As a consequence of using a fixed-effect framework we do not suggest that these results are generalizable to other studies not included in this review. A fixed-effect meta-analysis assumes that the sensitivity and specificity is homogenous across studies and so does not account for variability between studies. As a result, our pooled estimates will underestimate the uncertainty by failing to account for this variability. Although we did not account for between-study heterogeneity, we did account for within-study heterogeneity through the use of a fixed-effect conditional dependence structure between diagnostic tests in a study [35]. The small number of studies and limited data also prevented any further sub-analyses regarding the performance of the IMMY CrAg^®^ LFA between different patient groups or between different cryptococcal species.

Another limitation was that the quality assessment using the QUADAS-2 tool identified unclear or high risk of bias in all studies. A key concern was that reference standard tests were interpreted with prior knowledge of the result of the index test and that populations being tested had already been classified as having cryptococcal infection. The flow and timing of testing was also unclear in a number of studies, with a lack of information regarding exclusions.

The main strengths of this review are that this is the first review looking at CrAg LFA testing of participants without HIV. It is also novel in calculating a value for specificity, where the majority of studies included in the review have focussed on sensitivity estimates only.

## Conclusions

This review estimates a high sensitivity and specificity for IMMY CrAg^®^ LFA in HIV-negative populations, as previously described for PLWH. However, our review was limited by a small number of disparate studies reporting IMMY CrAg^®^ LFA testing on HIV-negative persons. Further studies using IMMY CrAg^®^ LFA on both symptomatic patients being evaluated for cryptococcal disease and asymptomatic screening cohorts in HIV-negative populations are required to better predict the diagnostic value of this test. This is important given the increasing proportion of HIV-negative patients with cryptococcal infection.

## Supplementary Information

**Additional file 1.** Search Methodology.

## Data Availability

Code used for meta-analysis is publicly available at: https://github.com/shk313/diagnostic-test-metaanalysis/tree/main/CrAg. Data included in analyses can be found in Tables 1 and 2.

## Abbreviations

CALAS: Cryptococcal Antigen Latex Agglutination System
CSF: Cerebrospinal fluid
CrAg: Cryptococcal antigen
EIA: Enzyme immunoassay
ELISA: Enzyme-linked immunosorbent assay
GXM: Glucuronoxylomannan
HIV: Human immunodeficiency virus
HSROC: Hierarchical Summary Receiver Operating Characteristic
LA: Latex agglutination
LFA: Lateral flow assay
MALDI-TOF: Matrix-assisted laser desorption ionization–time-of-flight mass spectrometry
PCR: Polymerase chain reaction
PLWH: People living with HIV
QUADAS: Quality assessment of diagnostic-accuracy studies
RCT: Randomised controlled trial
