# Supplementary material for: Diagnostic performance of the IMMY cryptococcal antigen lateral flow assay on serum and cerebrospinal fluid for diagnosis of cryptococcosis in HIV-negative patients: a systematic review
Source: BMC Infect Dis. 2023 Apr 6;23:209. doi: 10.1186/s12879-023-08135-w (PMC10080957; doi:10.1186/s12879-023-08135-w)
Supplement: Supplementary file 1 — Additional file 1. Search Methodology. [file 12879_2023_8135_MOESM1_ESM.pdf]

FIEBRE Reviews: Cryptococcal antigen testing using Immuno-Mycologics lateral flow immunoassay on serum. Search methodology

Table of Contents

1 Search methodology 2
2 Databases 2
2.1 Information management 3
3 Results 3
4 References 4
5 Appendix: Search strategies 4
5.1 Initial searches run in 2020 4
5.1.1 OvidSP Medline 4
5.1.2 OvidSP Embase 5
5.1.3 OvidSP Global Health 7
5.1.4 Wiley Cochrane CENTRAL database 8
5.1.5 Clarivate Analytics Web of Science 9
5.1.6 Ebsco Africa-Wide Information 10
5.1.7 Scopus 11
5.1.8 LILACS 12
5.1.9 Global Index Medicus 13

Jane Falconer
User Support Services Librarian
jane.falconer@lshtm.ac.uk

Document updated: 01 September 2021

5.2 Updated searches run in 2021 13
5.2.1 OvidSP Medline 13
5.2.2 OvidSP Embase 14
5.2.3 OvidSP Global Health 16
5.2.4 Wiley Cochrane CENTRAL database 17
5.2.5 Clarivate Analytics Web of Science 18
5.2.6 Ebsco Africa-Wide Information 19
5.2.7 Scopus 20
5.2.8 LILACS 21
5.2.9 Global Index Medicus 22

1 Search methodology

A draft search strategy was compiled in the OvidSP Medline database by an experienced information specialist (JF). The search strategy included strings of terms, synonyms and controlled vocabulary terms (where available) to reflect two concepts:

Concept 1: cryptococcus

Concept 2: Immuno-Mycologics lateral flow assay

As recommended in the Cochrane Handbook for Systematic Reviews of Diagnostic Test Accuracy, a specific filter to identify diagnostic test accuracy studies was not included.[1] Animal studies were excluded using the relevant section of the Cochrane RCT filter.[2] Results were limited to those published 2009-current. No other filters or limits were added. This search strategy was refined with the project team until the results retrieved reflected the scope of the project. The agreed OvidSP Medline search was adapted for each database to incorporate database-specific syntax and controlled vocabularies. Full details of the search strings used for each database can be found in the appendix.

2 Databases

The following bibliographic databases were searched on 17 September 2020 and then the search results were updated on 05 July 2021.

- OvidSP Medline ALL, 1946 to September 16, 2020, then 1946 to July 02, 2021.
- OvidSP Embase, 1947 to 2020 September 16, then 1947 to 2021 July 02.
- OvidSP Global Health, 1910 to 2020 week 37 then 1910 to 2021 week 26.
- Wiley Cochrane Central Register of Controlled Trials, Issue 9 of 12, September 2020 then Issue 7 of 12, July 2021.
- Clarivate Analytics Web of Science, Data last updated 2020-09-16 then 2021-07-04:
    - Science Citation Index-Expanded, 1970-present;

- Social Sciences Citation Index, 1970-present.

  Both databases searched simultaneously
- Elsevier Scopus, complete database.
- Ebsco Africa-Wide Information, complete database.
- WHO LILACS, complete database.
- WHO Global Index Medicus, complete database.

2.1 Information management

All citations identified by the searches run in 2020 were imported into EndNote X9 software. Duplicates were identified and removed using the method described on the London School of Hygiene & Tropical Medicine Library & Archives Service blog.[3] All results from the 2021 search were imported into the same EndNote X9 library and duplicates were identified and removed, leaving one copy of each item retrieved.

3 Results

A total of 847 results were retrieved by the search run in 2020. 994 were retrieved by the search run in 2021. Number of results pre-and post-deduplication are listed in the table below.

Database name	Total number of results retrieved in 2020	Total number of results retrieved in 2021	Number of results once duplicates removed from 2020 results	Number of new results found from 2021 update
Medline	127	150	127	8
Embase	268	302	157	38
Global Health	98	113	13	2
Africa-Wide Information	27	29	4	1
Cochrane Central Register of Controlled Trials	3	3	1	0
Global Index Medicus	8	10	2	0
Web of Science databases (both searched together)	141	184	39	24
Scopus	170	196	27	4
LILACS	5	7	3	0

**Total**	**847**	**994**	**373**	**77**

4 References

1. de Vet HCW, Eisinga A, Riphagen II, Aertgeerts B, Pewsner D. Searching for Studies. Cochrane Handbook for Systematic Reviews of Diagnostic Test Accuracy: The Cochrane Collaboration; 2008. Available from: https://methods.cochrane.org/sites/methods.cochrane.org.sdt/files/public/uploads/Chapter07-Searching-%28September-2008%29.pdf

2. Lefebvre C, Glanville J, Briscoe S, Littlewood A, Marshall C, Metzendorf MI, et al. Technical Supplement to Chapter 4: Searching for and selecting studies. In: Higgins JPT, Thomas J, Chandler J, Cumpston MS, Li T, Page MJ, et al., editors. Cochrane Handbook for Systematic Reviews of Interventions 6th ed: Cochrane; 2019. Available from: https://training.cochrane.org/handbook/version-6/chapter-4-tech-suppl.

3. Falconer J. Removing duplicates from an EndNote Library. Library & Archives Service Blog [Internet]: London School of Hygiene & Tropical Medicine. 2018. [cited 2020]. Available from: https://blogs.lshtm.ac.uk/library/2018/12/07/removing-duplicates-from-an-endnote-library/.

5 Appendix: Search strategies

This appendix provides full details of all search strings used for bibliographic databases, with dates and number of references returned and notes explaining any unusual search techniques or syntax. The EndNote X9 import order is provided, as the deduplication technique keeps the first uploaded copy of the reference by default.

In all searches, numbers in parentheses at the end of each row show the number of hits retrieved.

5.1 Initial searches run in 2020

5.1.1 OvidSP Medline

Database name	Medline ALL
Database platform	OvidSP
Dates of database coverage	1946 to September 16, 2020
Date searched	17 September 2020
Searched by	JF

Number of results	127
EndNote import order	1
Number of results once duplicates removed	127
Search strategy notes	Search lines ending in a '/' are subject heading searches.<br>Search lines beginning 'exp' are exploded subject heading searches.<br>Two-letter codes at the end of search lines designate the fields to search. Fields codes used are:<br>TI: title<br>AB: abstract<br>KF: author keywords<br>SH: subject heading<br>or/*x-y* combines search sets in the range *x-y* with Boolean operator OR.<br>* is used for truncation of words.

1. exp Cryptococcus/ (8746)
2. exp Cryptococcosis/ (9154)
3. (cryptococc* or CrAg or filobasidiella).ti,ab,kf. (16540)
4. or/1-3 (18588)
5. Immunoassay/ (29584)
6. (lateral flow immunoassay or lateral flow assay or lateral flow immunochromato* or lfa).ti,ab,kf. (7747)
7. (immuno-mycologic* or immunomycologic* or immy).ti,ab,kf. (63)
8. (lateral flow test* or lateral flow device* or lft or lfd).ti,ab,kf. (2249)
9. (dipstick or express test* or pen-side test* or quick test* or rapid test* or test strip*).ti,ab,kf. (11775)
10. or/5-9 (49728)
11. 4 and 10 (157)
12. limit 11 to yr="2009 -Current" (132)
13. exp animals/ not humans.sh. (4735057)
14. 12 not 13 (128)
15. remove duplicates from 14 (127)

5.1.2 OvidSP Embase

Database name	Embase Classic+Embase
Database platform	OvidSP

Dates of database coverage	1947 to 2020 September 16
Date searched	17 September 2020
Searched by	JF
Number of results	268
EndNote import order	2
Number of results once duplicates removed	157
Search strategy notes	Search lines ending in a '/' are subject heading searches.<br>Search lines beginning 'exp' are exploded subject heading searches.<br>Two-letter codes at the end of search lines designate the fields to search. Fields codes used are:<br>TI: title<br>AB: abstract<br>KW: author keywords<br>SH: subject heading<br>or/*x-y* combines search sets in the range *x-y* with Boolean operator OR.<br>* is used for truncation of words.

1. exp filobasidiella/ (13117)
2. exp cryptococcosis/ (11563)
3. (cryptococc* or CrAg or filobasidiella).ti,ab,kw. (19018)
4. or/1-3 (24106)
5. immunoassay/ (68049)
6. (lateral flow immunoassay or lateral flow assay or lateral flow immunochromato* or lfa).ti,ab,kw. (9243)
7. (immuno-mycologic* or immunomycologic* or immy).ti,ab,kw. (94)
8. (lateral flow test* or lateral flow device* or lft or lfd).ti,ab,kw. (4771)
9. (dipstick or express test* or pen-side test* or quick test* or rapid test* or test strip*).ti,ab,kw. (16861)
10. or/5-9 (96588)
11. 4 and 10 (318)
12. (rat or rats or mouse or mice or swine or porcine or murine or sheep or lambs or pigs or piglets or rabbit or rabbits or cat or cats or dog or dogs or cattle or bovine or monkey or monkeys or trout or marmoset$1).ti. and animal experiment/ (1077737)
13. Animal experiment/ not (human experiment/ or human/) (2270382)

14. 12 or 13 (2319920)
15. 11 not 14 (311)
16. limit 15 to yr="2009 -Current" (273)
17. remove duplicates from 16 (268)

5.1.3 OvidSP Global Health

Database name	Global Health
Database platform	OvidSP
Dates of database coverage	1910 to 2020 week 37
Date searched	17 September 2020
Searched by	JF
Number of results	98
EndNote import order	3
Number of results once duplicates removed	13
Search strategy notes	Search lines ending in a '/' are subject heading searches.<br>Search lines beginning 'exp' are exploded subject heading searches.<br>Two-letter codes at the end of search lines designate the fields to search. Fields codes used are:<br>TI: title<br>AB: abstract<br>SH: subject heading<br>or/*x-y* combines search sets in the range *x-y* with Boolean operator OR.<br>* is used for truncation of words.

1. exp cryptococcosis/ (9316)
2. exp "cryptococcus (fungi)"/ (15615)
3. exp filobasidiella/ (28)
4. (cryptococc* or CrAg or filobasidiella).ti,ab. (15362)
5. or/1-4 (16921)
6. immunoassay/ (5566)
7. (lateral flow immunoassay or lateral flow assay or lateral flow immunochromato* or lfa).ti,ab. (896)
8. (immuno-mycologic* or immunomycologic* or immy).ti,ab. (42)

9. (lateral flow test* or lateral flow device* or lft or lfd).ti,ab. (900)
10. (dipstick or express test* or pen-side test* or quick test* or rapid test* or test strip*).ti,ab. (5527)
11. or/6-10 (12222)
12. 5 and 11 (123)
13. ((rat or rats or mouse or mice or swine or porcine or murine or sheep or lambs or pigs or piglets or rabbit or rabbits or cat or cats or dog or dogs or cattle or bovine or monkey or monkeys or trout or marmoset$1) not man).sh. (506983)
14. 12 not 13 (121)
15. limit 14 to yr="2009 -Current" (98)
16. remove duplicates from 15 (98)

5.1.4 Wiley Cochrane CENTRAL database

Database name	Cochrane Central Register of Controlled Trials
Database platform	Wiley
Dates of database coverage	Issue 09 of 12, September 2020
Date searched	17 September 2020
Searched by	JF
Number of results	3
EndNote import order	7
Number of results once duplicates removed	1
Search strategy notes	* is used for truncation.<br>NEAR/n finds terms within n words of each other in any order.<br>NEXT finds terms next to each other in the order they have been entered. This is used for phrase searching where truncation is used.<br># is used for compulsory wildcards<br>? is used for optional wildcards<br>Searches ending :ti,ab,kw search the title, abstract and keywords.

	Note, numbers in parentheses are results across all Cochrane databases.

#1 MeSH descriptor: [Cryptococcus] explode all trees (24)
#2 MeSH descriptor: [Cryptococcosis] explode all trees (88)
#3 (cryptococc* or CrAg or filobasidiella):ti,ab,kw (283)
#4 #1 or #2 or #3 (283)
#5 MeSH descriptor: [Immunoassay] explode all trees (4559)
#6 ("lateral flow immunoassay" or "lateral flow assay" or "lateral flow immunochromato*" or fla):ti,ab,kw (121)
#7 ("immuno-mycologic*" or immunomycologic* or immy):ti,ab,kw (1)
#8 ("lateral flow test*" or "lateral flow device*" or lft or lfd):ti,ab,kw (503)
#9 (dipstick or "express test*" or "pen-side test*" or "quick test*" or "rapid test*" or "test strip*"):ti,ab,kw (947)
#10 #5 or #6 or #7 or #8 or #9 (6104)
#11 #4 and #10 (4)

5.1.5 Clarivate Analytics Web of Science

Database name	Science Citation Index Expanded<br>Social Sciences Citation Index
Database platform	Clarivate Analytics Web of Science
Dates of database coverage	Both databases 1970-present.<br>Data last updated 2020-09-16
Date searched	17 September 2020
Searched by	JF
Number of results	141
EndNote import order	5
Number of results once duplicates removed	39
Search strategy notes	* is used for truncation.<br>TOPIC and TS searches search in the title, abstract and keywords fields.<br>TI searches search in the title.

	All searches run across Indexes=SCI-EXPANDED, SSCI Timespan=2009-2020

#1 TOPIC: (cryptococc* or CrAg or filobasidiella) (9,209)
#2 TOPIC: ("lateral flow immunoassay" or "lateral flow assay" or "lateral flow immunochromato*" or lfa) (3,951)
#3 TOPIC: ("immuno-mycologic*" or immunomycologic* or immy) (33)
#4 TOPIC: ("lateral flow test*" or "lateral flow device*" or lft or lfd) (2,266)
#5 TOPIC: (dipstick or "express test*" or "pen-side test*" or "quick test*" or "rapid test*" or "test strip*") (8,320)
#6 #5 OR #4 OR #3 OR #2 (13,743)
#7 #6 AND #1 (143)
#8 TI=(rat or rats or mouse or mice or swine or porcine or murine or sheep or lambs or pigs or piglets or rabbit or rabbits or cat or cats or dog or dogs or cattle or bovine or monkey or monkeys or trout or marmoset*) NOT TS=(man or human) (628,142)
#9 #7 not #8 (141)

5.1.6 Ebsco Africa-Wide Information

Database name	Africa-Wide Information
Database platform	Ebsco
Dates of database coverage	Complete database as of search date
Date searched	17 September 2020
Searched by	JF
Number of results	27
EndNote import order	4
Number of results once duplicates removed	4
Search strategy notes	Two-letter codes at the beginning of search lines designate the fields to search. Fields codes used are:<br>TI: title<br>AB: abstract<br>KW: keywords<br>* is used for truncation of words.<br>N*n* searches for terms within *n* words of each other.

S1 (TI (cryptococc* or CrAg or filobasidiella)) or (AB (cryptococc* or CrAg or filobasidiella)) or (KW (cryptococc* or CrAg or filobasidiella)) (2,576)

S2 (TI ("lateral flow immunoassay" or "lateral flow assay" or "lateral flow immunochromato* or lfa)) or (AB ("lateral flow immunoassay" or "lateral flow assay" or "lateral flow immunochromato* or lfa)) or (KW ("lateral flow immunoassay" or "lateral flow assay" or "lateral flow immunochromato* or lfa)) (84)

S3 (TI ("immuno-mycologic*" or immunomycologic* or immy)) or (AB ("immuno-mycologic*" or immunomycologic* or immy)) or (KW ("immuno-mycologic*" or immunomycologic* or immy)) (11)

S4 (TI ("lateral flow test*" or "lateral flow device*" or lft or lfd)) or (AB ("lateral flow test*" or "lateral flow device*" or lft or lfd)) or (KW ("lateral flow test*" or "lateral flow device*" or lft or lfd)) (147)

S5 (TI (dipstick or "express test*" or "pen-side test*" or "quick test*" or "rapid test*" or "test strip*")) or (AB (dipstick or "express test*" or "pen-side test*" or "quick test*" or "rapid test*" or "test strip*")) or (KW (dipstick or "express test*" or "pen-side test*" or "quick test*" or "rapid test*" or "test strip*")) (2,048)

S6 S2 OR S3 OR S4 OR S5 (2,247)

S7 S1 AND S6 (28)

S8 (TI (rat or rats or mouse or mice or swine or porcine or murine or sheep or lambs or pigs or piglets or rabbit or rabbits or cat or cats or dog or dogs or cattle or bovine or monkey or monkeys or trout or marmoset*)) 83,660

S9 (TI (man or human)) OR (AB (man or human)) OR (KW (man or human)) (513,494)

S10 S8 not S9 (73,693)

S11 S7 not S10 (28)

S12 S11 Limiters - Year Published: 2009-2020 (27)

5.1.7 Scopus

Database name	Scopus
Database platform	Scopus.com
Dates of database coverage	Complete database as of search date
Date searched	17 September 2020
Searched by	JF
Number of results	170
EndNote import order	6
Number of results once duplicates removed	27

Search strategy notes	* is used for truncation of words.<br>{ } searches for exact term with no lemmatization or stemming.

((TITLE-ABS-KEY (cryptococc* OR crag OR filobasidiella)) AND (TITLE-ABS-KEY ("lateral flow immunoassay" OR "lateral flow assay" OR "lateral flow immunochromato*" OR lfa OR "immuno-mycologic*" OR immunomycologic* OR immy OR "lateral flow test*" OR "lateral flow device*" OR flt OR lfd OR dipstick OR "express test*" OR "pen-side test*" OR "quick test*" OR "rapid test*" OR "test strip*"))) AND NOT ((TITLE ({rat} OR {rats} OR {mouse} OR {mice} OR {swine} OR {porcine} OR {murine} OR {sheep} OR {lambs} OR {pigs} OR {piglets} OR {rabbit} OR {rabbits} OR {cat} OR {cats} OR {dog} OR {dogs} OR {cattle} OR {bovine} OR {monkey} OR {monkeys} OR {trout} OR marmoset*)) AND NOT (TITLE-ABS-KEY ({man} OR {human})))) AND (LIMIT-TO (PUBYEAR , 2020) OR LIMIT-TO (PUBYEAR , 2019) OR LIMIT-TO (PUBYEAR , 2018) OR LIMIT-TO (PUBYEAR , 2017) OR LIMIT-TO (PUBYEAR , 2016) OR LIMIT-TO (PUBYEAR , 2015) OR LIMIT-TO (PUBYEAR , 2014) OR LIMIT-TO (PUBYEAR , 2013) OR LIMIT-TO (PUBYEAR , 2012) OR LIMIT-TO (PUBYEAR , 2011) OR LIMIT-TO (PUBYEAR , 2010) OR LIMIT-TO (PUBYEAR , 2009))

5.1.8 LILACS

Database name	LILACS
Database platform	WHO Global Index Medicus
Dates of database coverage	Complete database as of search date
Date searched	17 September 2020
Searched by	JF
Number of results	5
EndNote import order	8
Number of results once duplicates removed	3
Search strategy notes	* is used for truncation of words.

(tw:(cryptococc* or CrAg or filobasidiella)) AND (tw:("lateral flow immunoassay" or "lateral flow assay" or "lateral flow immunochromatographic" or "lateral flow immunochromatography" or lfa or "immuno-mycologics" or immy or "lateral flow test" or "lateral flow tests" or "lateral flow testing" or "lateral flow device" or "lateral flow devices"

or lft or lfd or dipstick or "express test" or "express tests" or "express testing" or "pen-side test" or "pen-side tests" or "pen-side testing" or "quick test" or "quick tests" or "quick testing" or "rapid test" or "rapid tests" or "rapid testing" or "test strip" or "test strips"))

5.1.9 Global Index Medicus

Database name	Global Index Medicus
Database platform	WHO Global Index Medicus
Dates of database coverage	Complete database as of search date
Date searched	17 September 2020
Searched by	JF
Number of results	8
EndNote import order	9
Number of results once duplicates removed	2
Search strategy notes	* is used for truncation of words.

(tw:(cryptococc* or CrAg or filobasidiella)) AND (tw:("lateral flow immunoassay" or "lateral flow assay" or "lateral flow immunochromatographic" or "lateral flow immunochromatography" or lfa or "immuno-mycologics" or immy or "lateral flow test" or "lateral flow tests" or "lateral flow testing" or "lateral flow device" or "lateral flow devices" or lft or lfd or dipstick or "express test" or "express tests" or "express testing" or "pen-side test" or "pen-side tests" or "pen-side testing" or "quick test" or "quick tests" or "quick testing" or "rapid test" or "rapid tests" or "rapid testing" or "test strip" or "test strips"))

5.2 Updated searches run in 2021

5.2.1 OvidSP Medline

Database name	Medline ALL
Database platform	OvidSP
Dates of database coverage	1946 to July 02, 2021
Date searched	05 July 2021

Searched by	JF
Number of results	150
EndNote import order	1
Number of results once duplicates removed	8
Search strategy notes	Search lines ending in a '/' are subject heading searches.<br>Search lines beginning 'exp' are exploded subject heading searches.<br>Two-letter codes at the end of search lines designate the fields to search. Fields codes used are:<br>TI: title<br>AB: abstract<br>KF: author keywords<br>SH: subject heading<br>or/*x*-*y* combines search sets in the range *x*-*y* with Boolean operator OR.<br>* is used for truncation of words.

1 exp Cryptococcus/ (9019)
2 exp Cryptococcosis/ (9446)
3 (cryptococc* or CrAg or filobasidiella).ti,ab,kf. (17124)
4 or/1-3 (19195)
5 Immunoassay/ (31144)
6 (lateral flow immunoassay or lateral flow assay or lateral flow immunochromato* or lfa).ti,ab,kf. (8252)
7 (immuno-mycologic* or immunomycologic* or immy).ti,ab,kf. (80)
8 (lateral flow test* or lateral flow device* or lft or lfd).ti,ab,kf. (2561)
9 (dipstick or express test* or pen-side test* or quick test* or rapid test* or test strip*).ti,ab,kf. (12744)
10 or/5-9 (52680)
11 4 and 10 (180)
12 limit 11 to yr="2009 -Current" (155)
13 exp animals/ not humans.sh. (4855957)
14 12 not 13 (151)
15 remove duplicates from 14 (150)

5.2.2 OvidSP Embase

Database name	Embase Classic+Embase

Database platform	OvidSP
Dates of database coverage	1947 to 2021 July 02
Date searched	05 July 2021
Searched by	JF
Number of results	302
EndNote import order	2
Number of results once duplicates removed	38
Search strategy notes	Search lines ending in a '/' are subject heading searches.<br>Search lines beginning 'exp' are exploded subject heading searches.<br>Two-letter codes at the end of search lines designate the fields to search. Fields codes used are:<br>TI: title<br>AB: abstract<br>KW: author keywords<br>SH: subject heading<br>or/*x-y* combines search sets in the range *x-y* with Boolean operator OR.<br>* is used for truncation of words.

1. exp filobasidiella/ (14967)
2. exp cryptococcosis/ (13598)
3. (cryptococc* or CrAg or filobasidiella).ti,ab,kw. (21503)
4. or/1-3 (27810)
5. immunoassay/ (74004)
6. (lateral flow immunoassay or lateral flow assay or lateral flow immunochromato* or lfa).ti,ab,kw. (9797)
7. (immuno-mycologic* or immunomycologic* or immy).ti,ab,kw. (113)
8. (lateral flow test* or lateral flow device* or lft or lfd).ti,ab,kw. (5337)
9. (dipstick or express test* or pen-side test* or quick test* or rapid test* or test strip*).ti,ab,kw. (18937)
10. or/5-9 (105374)
11. 4 and 10 (357)

12. (rat or rats or mouse or mice or swine or porcine or murine or sheep or lambs or pigs or piglets or rabbit or rabbits or cat or cats or dog or dogs or cattle or bovine or monkey or monkeys or trout or marmoset$1).ti. and animal experiment/ (1112835)
13. Animal experiment/ not (human experiment/ or human/) (2338707)
14. 12 or 13 (2393366)
15. 11 not 14 (349)
16. limit 15 to yr="2009 -Current" (308)
17. remove duplicates from 16 (302)

5.2.3 OvidSP Global Health

Database name	Global Health
Database platform	OvidSP
Dates of database coverage	1910 to 2021 week 26
Date searched	05 July 2021
Searched by	JF
Number of results	113
EndNote import order	3
Number of results once duplicates removed	4
Search strategy notes	Search lines ending in a ‘/’ are subject heading searches.<br>Search lines beginning ‘exp’ are exploded subject heading searches.<br>Two-letter codes at the end of search lines designate the fields to search. Fields codes used are:<br>TI: title<br>AB: abstract<br>SH: subject heading<br>or/*x*-*y* combines search sets in the range *x*-*y* with Boolean operator OR.<br>* is used for truncation of words.

1 exp cryptococcosis/ (9510)
2 exp "cryptococcus (fungi)"/ (16020)
3 exp filobasidiella/ (28)
4 (cryptococc* or CrAg or filobasidiella).ti,ab. (15741)
5 or/1-4 (17331)

6 immunoassay/ (6178)
7 (lateral flow immunoassay or lateral flow assay or lateral flow immunochromato* or lfa).ti,ab. (1059)
8 (immuno-mycologic* or immunomycologic* or immy).ti,ab. (51)
9 (lateral flow test* or lateral flow device* or lft or lfd).ti,ab. (1011)
10 (dipstick or express test* or pen-side test* or quick test* or rapid test* or test strip*).ti,ab. (5955)
11 or/6-10 (13404)
12 5 and 11 (138)
13 ((rat or rats or mouse or mice or swine or porcine or murine or sheep or lambs or pigs or piglets or rabbit or rabbits or cat or cats or dog or dogs or cattle or bovine or monkey or monkeys or trout or marmoset$1) not man).sh. (522300)
14 12 not 13 (136)
15 limit 14 to yr="2009 -Current" (113)
16 remove duplicates from 15 (113)

5.2.4 Wiley Cochrane CENTRAL database

Database name	Cochrane Central Register of Controlled Trials
Database platform	Wiley
Dates of database coverage	Issue 07 of 12, July 2021
Date searched	05 July 2021
Searched by	JF
Number of results	3
EndNote import order	7
Number of results once duplicates removed	0
Search strategy notes	* is used for truncation.<br>NEAR/n finds terms within n words of each other in any order.<br>NEXT finds terms next to each other in the order they have been entered. This is used for phrase searching where truncation is used.<br># is used for compulsory wildcards

	? is used for optional wildcards<br><br>Searches ending :ti,ab,kw search the title, abstract and keywords.<br><br>Note, numbers in parentheses are results across all Cochrane databases.

#1 MeSH descriptor: [Cryptococcus] explode all trees (25)
#2 MeSH descriptor: [Cryptococcosis] explode all trees (90)
#3 (cryptococc* or CrAg or filobasidiella):ti,ab,kw (301)
#4 #1 OR #2 OR #3 (301)
#5 MeSH descriptor: [Immunoassay] explode all trees (4585)
#6 ("lateral flow immunoassay" or "lateral flow assay" or "lateral flow immunochromato*" or fla):ti,ab,kw (131)
#7 ("immuno-mycologic*" or immunomycologic* or immy):ti,ab,kw (1)
#8 ("lateral flow test*" or "lateral flow device*" or lft or lfd):ti,ab,kw (593)
#9 (dipstick or "express test*" or "pen-side test*" or "quick test*" or "rapid test*" or "test strip*"):ti,ab,kw (1066)
#10 #5 OR #6 OR #7 OR #8 OR #9 (6346)
#11 #4 AND #10 (4)

5.2.5 Clarivate Analytics Web of Science

Database name	Science Citation Index Expanded<br>Social Sciences Citation Index
Database platform	Clarivate Analytics Web of Science
Dates of database coverage	Both databases 1970-present.<br>Data last updated 2021-07-04
Date searched	05 July 2021
Searched by	JF
Number of results	184
EndNote import order	5
Number of results once duplicates removed	24
Search strategy notes	* is used for truncation.

	TOPIC and TS searches search in the title, abstract and keywords fields.<br><br>TI searches search in the title.<br><br>All searches run across Indexes=SCI-EXPANDED, SSCI Timespan=2009-2020

1 TOPIC: (cryptococc* or CrAg or filobasidiella) (19,367)
2 TOPIC: ("lateral flow immunoassay" or "lateral flow assay" or "lateral flow immunochromato*" or lfa) (10,906)
3 TOPIC: ("immuno-mycologic*" or immunomycologic* or immy) (66)
4 TOPIC: ("lateral flow test*" or "lateral flow device*" or lft or lfd) (3,259)
5 TOPIC: (dipstick or "express test*" or "pen-side test*" or "quick test*" or "rapid test*" or "test strip*") (14,476)
6 #5 OR #4 OR #3 OR #2 (27,646)
7 #6 AND #1 (184)

5.2.6 Ebsco Africa-Wide Information

Database name	Africa-Wide Information
Database platform	Ebsco
Dates of database coverage	Complete database as of search date
Date searched	05 July 2021
Searched by	JF
Number of results	29
EndNote import order	4
Number of results once duplicates removed	1
Search strategy notes	Two-letter codes at the beginning of search lines designate the fields to search. Fields codes used are:<br>TI: title<br>AB: abstract<br>KW: keywords<br>* is used for truncation of words.<br>N*n* searches for terms within *n* words of each other.

S1 (TI (cryptococc* or CrAg or filobasidiella)) or (AB (cryptococc* or CrAg or filobasidiella)) or (KW (cryptococc* or CrAg or filobasidiella)) (2,586)

S2 (TI ("lateral flow immunoassay" or "lateral flow assay" or "lateral flow immunochromato* or lfa)) or (AB ("lateral flow immunoassay" or "lateral flow assay" or "lateral flow immunochromato* or lfa)) or (KW ("lateral flow immunoassay" or "lateral flow assay" or "lateral flow immunochromato* or lfa)) (87)

S3 (TI ("immuno-mycologic*" or immunomycologic* or immy)) or (AB ("immuno-mycologic*" or immunomycologic* or immy)) or (KW ("immuno-mycologic*" or immunomycologic* or immy)) (11)

S4 (TI ("lateral flow test*" or "lateral flow device*" or lft or lfd)) or (AB ("lateral flow test*" or "lateral flow device*" or lft or lfd)) or (KW ("lateral flow test*" or "lateral flow device*" or lft or lfd)) (147)

S5 (TI (dipstick or "express test*" or "pen-side test*" or "quick test*" or "rapid test*" or "test strip*")) or (AB (dipstick or "express test*" or "pen-side test*" or "quick test*" or "rapid test*" or "test strip*")) or (KW (dipstick or "express test*" or "pen-side test*" or "quick test*" or "rapid test*" or "test strip*")) (2,083)

S6 S2 OR S3 OR S4 OR S5 (2,285)

S7 S1 AND S6 (30)

S8 (TI (rat or rats or mouse or mice or swine or porcine or murine or sheep or lambs or pigs or piglets or rabbit or rabbits or cat or cats or dog or dogs or cattle or bovine or monkey or monkeys or trout or marmoset*)) (84,154)

S9 (TI (man or human)) OR (AB (man or human)) OR (KW (man or human)) (515,599)

S10 S8 NOT S9 (74,151)

S11 S7 NOT S10 (30)

S12 S11 Limiters - Year Published: 2009-2021 (29)

5.2.7 Scopus

Database name	Scopus
Database platform	Scopus.com
Dates of database coverage	Complete database as of search date
Date searched	05 July 2021
Searched by	JF
Number of results	196
EndNote import order	6
Number of results once duplicates removed	4

Search strategy notes	* is used for truncation of words.<br>{ } searches for exact term with no lemmatization or stemming.

((TITLE-ABS-KEY (cryptococc* OR crag OR filobasidiella)) AND (TITLE-ABS-KEY ("lateral flow immunoassay" OR "lateral flow assay" OR "lateral flow immunochromato*" OR lfa OR "immuno-mycologic*" OR immunomycologic* OR immy OR "lateral flow test*" OR "lateral flow device*" OR flt OR lfd OR dipstick OR "express test*" OR "pen-side test*" OR "quick test*" OR "rapid test*" OR "test strip*"))) AND NOT ((TITLE ({rat} OR {rats} OR {mouse} OR {mice} OR {swine} OR {porcine} OR {murine} OR {sheep} OR {lambs} OR {pigs} OR {piglets} OR {rabbit} OR {rabbits} OR {cat} OR {cats} OR {dog} OR {dogs} OR {cattle} OR {bovine} OR {monkey} OR {monkeys} OR {trout} OR marmoset*)) AND NOT (TITLE-ABS-KEY ({man} OR {human})))) AND (LIMIT-TO (PUBYEAR , 2021) OR (LIMIT-TO (PUBYEAR , 2020) OR LIMIT-TO (PUBYEAR , 2019) OR LIMIT-TO (PUBYEAR , 2018) OR LIMIT-TO (PUBYEAR , 2017) OR LIMIT-TO (PUBYEAR , 2016) OR LIMIT-TO (PUBYEAR , 2015) OR LIMIT-TO (PUBYEAR , 2014) OR LIMIT-TO (PUBYEAR , 2013) OR LIMIT-TO (PUBYEAR , 2012) OR LIMIT-TO (PUBYEAR , 2011) OR LIMIT-TO (PUBYEAR , 2010) OR LIMIT-TO (PUBYEAR , 2009))

5.2.8 LILACS

Database name	LILACS
Database platform	WHO Global Index Medicus
Dates of database coverage	Complete database as of search date
Date searched	05 July 2021
Searched by	JF
Number of results	7
EndNote import order	8
Number of results once duplicates removed	0
Search strategy notes	* is used for truncation of words.

(tw:(cryptococc* or CrAg or filobasidiella)) AND (tw:("lateral flow immunoassay" or "lateral flow assay" or "lateral flow immunochromatographic" or "lateral flow immunochromatography" or lfa or "immuno-mycologics" or immy or "lateral flow test" or "lateral flow tests" or "lateral flow testing" or "lateral flow device" or "lateral flow devices"

or lft or lfd or dipstick or "express test" or "express tests" or "express testing" or "pen-side test" or "pen-side tests" or "pen-side testing" or "quick test" or "quick tests" or "quick testing" or "rapid test" or "rapid tests" or "rapid testing" or "test strip" or "test strips"))

5.2.9 Global Index Medicus

Database name	Global Index Medicus
Database platform	WHO Global Index Medicus
Dates of database coverage	Complete database as of search date
Date searched	05 July 2021
Searched by	JF
Number of results	10
EndNote import order	9
Number of results once duplicates removed	0
Search strategy notes	* is used for truncation of words.

(tw:(cryptococc* or CrAg or filobasidiella)) AND (tw:("lateral flow immunoassay" or "lateral flow assay" or "lateral flow immunochromatographic" or "lateral flow immunochromatography" or lfa or "immuno-mycologics" or immy or "lateral flow test" or "lateral flow tests" or "lateral flow testing" or "lateral flow device" or "lateral flow devices" or lft or lfd or dipstick or "express test" or "express tests" or "express testing" or "pen-side test" or "pen-side tests" or "pen-side testing" or "quick test" or "quick tests" or "quick testing" or "rapid test" or "rapid tests" or "rapid testing" or "test strip" or "test strips"))
